# Antitumor Activity of cGAMP *via* Stimulation of cGAS-cGAMP-STING-IRF3 Mediated Innate Immune Response

**DOI:** 10.1038/srep19049

**Published:** 2016-01-12

**Authors:** Tiejun Li, Hao Cheng, Hong Yuan, Qiming Xu, Chang Shu, Yuefan Zhang, Pengbiao Xu, Jason Tan, Yaocheng Rui, Pingwei Li, Xiangshi Tan

**Affiliations:** 1 Department of Chemistry & Shanghai Key Laboratory of Chemical Biology for Protein Research and Institutes of Biomedical Sciences, Fudan University, Shanghai, 200433, China; 2 Department of Pharmacology, School of Pharmacy, Second Military Medical University, Shanghai, 200433, China; 3 Department of Biochemistry and Biophysics, Texas A&M University, College Station, TX 77843, USA

## Abstract

Immunotherapy is one of the key strategies for cancer treatment. The cGAS-cGAMP-STING-IRF3 pathway of cytosolic DNA sensing plays a pivotal role in antiviral defense. We report that the STING activator cGAMP possesses significant antitumor activity in mice by triggering the STING-dependent pathway directly. cGAMP enhances innate immune responses by inducing production of cytokines such as interferon-β, interferon-γ, and stimulating dendritic cells activation, which induces the cross-priming of CD8^+^ T cells. The antitumor mechanism of cGAMP was verified by STING and IRF3, which were up-regulated upon cGAMP treatment. STING-deficiency dramatically reduced the antitumor effect of cGAMP. Furthermore, cGAMP improved the antitumor activity of 5-FU, and clearly reduced the toxicity of 5-FU. These results demonstrated that cGAMP is a novel antitumor agent and has potential applications in cancer immunotherapy.

Cancer is one of the most frequent causes of death worldwide despite significant improvements in cancer therapies during the past decades. Immunotherapy provides renovating treatment of cancer patients, and is becoming one of the key strategies to cure cancer^1^. Although cancer immunotherapy exhibits promising perspective, and is milder and more manageable than traditional or targeted cancer therapy, the molecular mechanism for tumor immunogenicity is usually unclear. Therefore, cancer immunotherapy strategies are still limited. It has been suggested that the optimal cancer immunotherapeutics will come from a combination of therapeutic strategies involving the manipulation of innate immunity, which plays a critical role in promoting T cell mediated immune response to cancer^2^. Immunotherapy is the most effective when an immune response is already underway, with activated T cells and dying tumor cells^3^. The generation of immunity to cancer is a cyclic process that can be self-propagating, leading to an accumulation of immune-stimulatory factors that in principle should amplify and broaden T cell response^1^,^2^,^4^. Induction of potent tumor-specific cytotoxic T-cell responses is one set of fundamental objectives in anticancer therapeutic strategies^5^. This event requires that antigen-presenting cells (APCs) present tumor-associated antigens (Ag) on their MHC class-I molecule (cross-presentation) to stimulate native CD8^+^ T cells (a process termed cross-priming). Dendritic cells (DCs) are the most important APCs which present tumor derived antigens *in vivo*^6^,^7^. DCs are particularly keen on this task and can induce the cross-priming of CD8^+^ T cells, when they are exposed to danger or inflammatory signals that stimulate their activation^8^. Recently, *in vitro* and *in vivo* studies have suggested that Type-I interferon (IFN), including IFN-α and IFN-β, stimulates cross-priming by DC against tumor-associated antigens is a key mechanism for cancer immune surveillance and can be targeted to boost anti-tumor CD8^+^ T-cell responses^8^.

Recent investigations have identified cyclic GMP-AMP synthase (cGAS) as a key cytosolic DNA sensor in innate immunity^9^. cGAS is activated by double strand DNA (dsDNA) and catalyzes the synthesis of a non-canonical cyclic dinucleotide 2′, 3′ cGAMP (cGAMP)^10^,^11^,^12^,^13^. cGAMP serves as a second messenger to induce the production of IFN-β and other cytokines via the adaptor protein stimulator of interferon genes complex (STING) locating in the ER membrane^14^. cGAMP induces a conformational change of STING, leading to the recruitment and activation of protein kinase TBK1 at the signaling complex. The transcription factor interferon regulatory factor 3 (IRF3) is then recruited to the signaling complex and phosphorylated by TBK1^11^,^15^. Phosphorylated IRF3 initiates the expression of IFN-β, which regulates the expression of over two hundred interferon-inducible genes that can down regulate protein synthesis, induce cell growth arrest and apoptosis, thus creating an antiviral effect^15^,^16^,^17^. Recent studies showed that STING-dependent cytosolic DNA sensing can mediate innate immune recognition of immunogenic tumors, and promote type-I IFN dependent antitumor immunity after radiation therapy^18^,^19^. IFN-γ can also exert antitumor effect *in vivo* via the tumor necrosis factor-related apoptosis-inducing ligand (TRAIL) pathway, which causes apoptosis of tumor cells^20^. The host STING pathway can play a critical role in the therapeutic efficacy of cancer immunotherapies. To understand the structural basis for cGAMP to activate STING, the crystal structures of cGAS bound to dsDNA and STING bound to cGAMP have recently been determined respectively^21^,^22^,^23^,^24^,^25^,^26^. A novel cGAS-cGAMP-STING-IRF3 pathway of cytosolic sensing and signaling has been verified to mediate innate immune response, and plays pivotal roles in antiviral defense^27^. Viruses can transfer the antiviral second messenger cGAMP between cells^28^. The transfer of cGAMP by viruses may represent a defense mechanism to propagate immune responses to uninfected target cells^29^. cGAMP is a critical stimulator of cGAS-cGAMP-STING-IRF3-mediated innate immune responses and is a high affinity endogenous activator of STING, we reasoned that cGAMP should also promote antitumor immune responses in addition to its antiviral activity. We synthesized cGAMP using recombinant cGAS enzyme, and purified it by ion exchange chromatography to over 95% purity. And then, we herein systematically investigated the antitumor activity of cGAMP against murine Colon 26 adenocarcinoma in mouse models. Our results showed that cGAMP has potent antitumor activity *in vivo*. cGAMP induces the expression of a variety of cytokines and stimulate the activation of DCs. In addition, cGAMP also improves the antitumor activity of 5-FU and reduces the toxic side effects when used in combination with 5-FU. Our results established a solid basis for the development of cGAMP as a potential new antitumor immunotherapy drug.

## Results

### cGAMP Possesses Potent Antitumor Activity against Murine Colon 26 Adenocarcinoma

**Figure 1:**
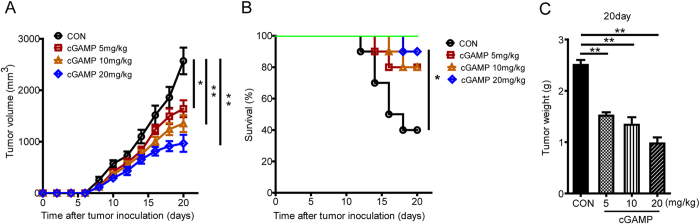
cGAMP Possesses Potent Anti-tumor Activity against Murine Colon 26 Adenocarcinoma. Colon 26 tumor-bearing mice were treated with cGAMP from day 0 to day 20 with 5, 10 and 20 mg/kg, respectively. (**A**) Mean tumor volumes. (**B**) Survival rates of mice. (**C**) Tumor weights. Representative data are shown from three experiments conducted with 10 mice per group. Data are represented as mean ± SEM, *p < 0.05 and **p < 0.01 (Student’s t test in **A** and **C**, and log rank [Mantel-Cox] test in **B**).

It has been demonstrated that cGAMP is an endogenous activator of STING and it is generated in mammalian cells by cGAS in response to double-strand DNA in the cytoplasm^13^,^19^,^30^. We hypothesized that exogenous cGAMP treatment might potentiate the antitumor effect by enhancing STING activation. To test this hypothesis, colon 26 cell suspensions were injected subcutaneously into the right flank of mice to develop tumor-bearing mice, which were treated with cGAMP daily at dosages of 5, 10 and 20 mg/kg in different group mice for 20 days. Tumor volumes and weights of mice were measured daily. On the 20^th^ day, all mice were weighed and sacrificed, and then tumors were isolated and measured. As shown in Fig. 1A,B, the tumor growth was effectively inhibited by cGAMP treatment from 2569 mm^3^ to 967 mm^3^ and the mice survival rates were obviously increased from 40% up to 90% for 20 days. The mean tumor weight was significantly decreased from 2.5 g to 0.97 g in a dose-dependent manner by cGAMP treatment (Fig. 1C). These results showed that cGAMP possessed potent antitumor activity against murine Colon 26 adenocarcinoma, and the antitumor activity was dose-dependent. Deng, *et al.* reported activation of STING by a second messenger cGAMP administration enhanced antitumor immunity induced by radiation^19^. However, they did not find the antitumor activity of cGAMP alone without radiation. The unsuccessful antitumor activity of cGAMP alone is likely due to low cGAMP dosage (0.5 mg/kg) and less administration times (only two times), compared with the amount of cGAMP (>5 mg/kg) and administration for 20 days in our experiment.

### cGAMP Induces Apoptosis of Tumor Cells

**Figure 2:**
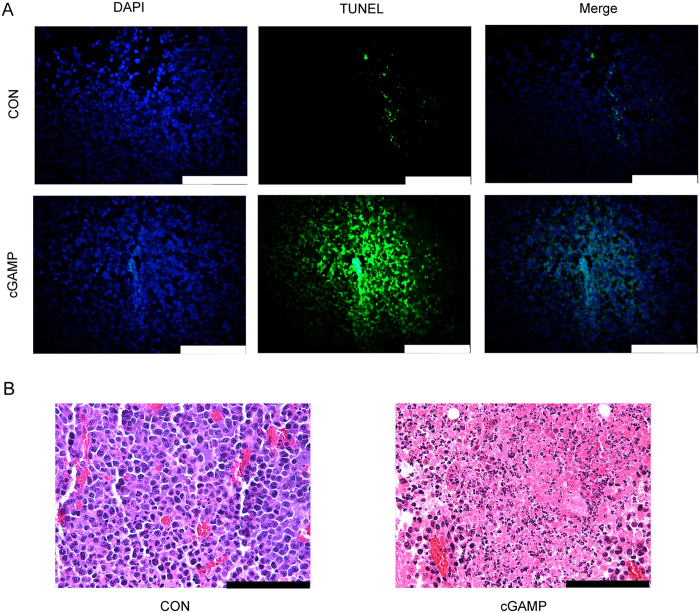
cGAMP Induces Apoptosis of Tumors. (**A**) TUNEL assay of tumor tissues using the “In Suit Cell Death Detection Kit, Fluorescein” (Roche, Germany). (**B**) Hematoxylin-Eosin staining assay of tumor tissues in control group and cGAMP-treated tumor-bearing mice (20 mg/kg). The nuclei and their fragments are blue-violet and the cytoplasm is pink-red. Scale bar = 100 μm. DAPI is 4,6-diamidino-2-phenylindole and TUNEL assay is terminal deoxynucleotidyl transferase-mediated dUTP nick end labeling assay.

To understand how cGAMP suppressed tumor growth and increased survive rates of tumor-bearing mice, we examined apoptosis in tumor tissues. The tumor damage in the tumor-bearing mice, after cGAMP treatment (20 mg/kg) for 20 days, was determined by terminal deoxynucleotidyl transferase-mediated deoxyuridine triphosphate nick end labeling (TUNEL) staining and Hematoxylin-Eosin (HE) staining assays. Compared to the control group, severe cell apoptosis occurred in the tumor tissues upon cGAMP treatment as indicated by TUNEL staining (Fig. 2A). In the HE staining assay, the nuclei and their fragments of tumor cells are blue-violet and the cytoplasm is pink-red (Fig. 2B). The tumor tissues in control mice displayed typical tumor tissue pattern with blue-violet color when treated with cGAMP, and the tumor tissues changed clearly from blue-violet to pink-red. These results demonstrated that the therapeutic effect of cGAMP is due to the induction of tumor cells apoptosis. To investigate whether cGAMP can kill tumor cells directly, cytotoxicity of cGAMP on CT26 cells were performed by MTT assay. As shown in Figure S1, cGAMP (10–200 μM) did not show obvious toxicity against CT26 cells and could not kill tumor cells directly *in vitro*. To investigate whether cGAMP induces apoptosis selectively in tumor cells, CD45 co-staining was preformed, and the results indicated that cGAMP selectively induced apoptosis of tumor cells while showing no impact on immune cells (Figure S2). Considering the role of cGAMP in STING pathway, we proposed that cGAMP might activate anti-tumor immunity through cGAS-cGAMP-STING-IRF3 innate immune pathway.

### cGAMP Induces the Expression of Cytokines in Mice

**Figure 3:**
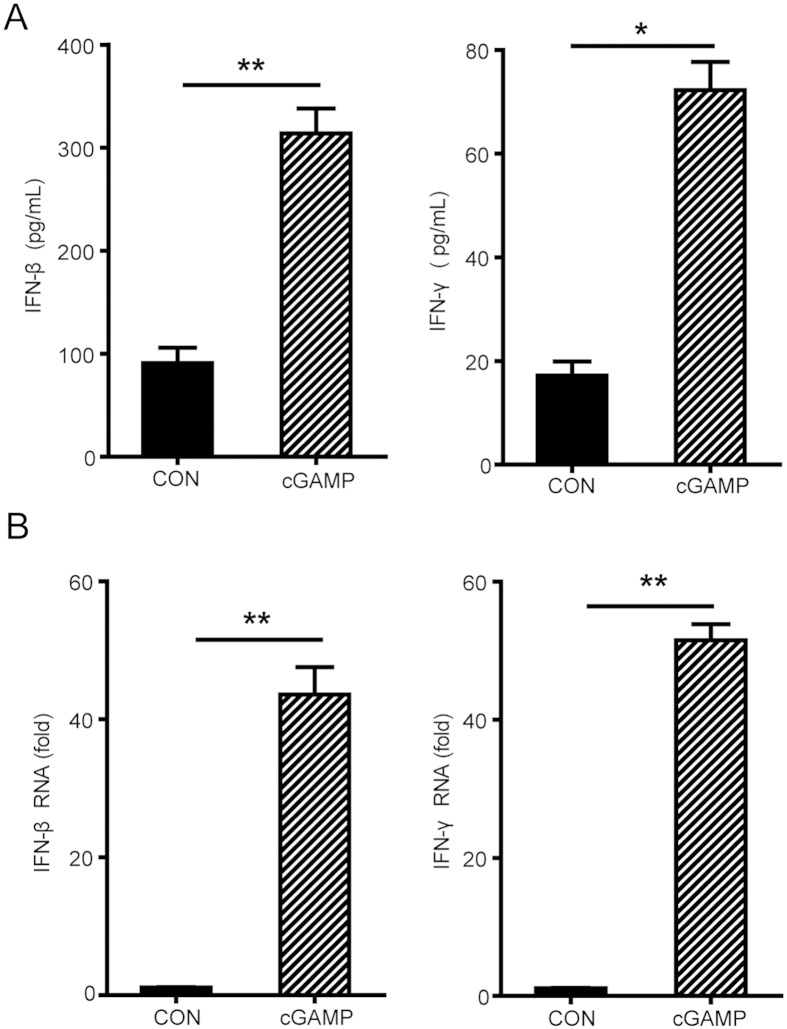
cGAMP Stimulates Cytokines Production. Colon 26 tumor-bearing mice were treated from day 0 to day 20 with 20 mg/kg cGAMP. (**A**) Cytokines production of IFN-β, IFN-γ were measured by ELISA in serum at the 20^th^ day and (**B**) Tumor tissues from tumor-bearing mice were detected by quantitative SYBR Green real-time PCR. Representative data are shown from three experiments conducted with 10 mice per group. Data are represented as mean ± SEM, *p < 0.05 and **p < 0.01(Student’s t test).

To test whether cGAMP stimulates innate immune responses and triggers antitumor cytokines production, we examined the induction of cytokines, including IFN-β, IFN-γ and other cytokines in the tumor-bearing mice. We observed that cGAMP up-regulated the expression levels of IFN-β and IFN-γ in the serum (Fig. 3A) and the mRNA levels of these cytokines in the tumor tissues (Fig. 3B). It has been shown that cGAMP serves as a second messenger to stimulate the production of IFN-β and other cytokines by direct binding to the adaptor protein STING in the innate immune pathway^9^,^25^. IFN-β production and dendritic cell activation may be triggered via the cGAS-cGAMP-STING-IRF3 innate immune pathway by cGAMP^21^,^22^. Diamond, *et al.* has shown that endogenously produced IFN-β is critical for the induction of an antitumor immune response resulting in the elimination of those tumors^31^. Targeting low doses of type-I IFNs to the tumor microenvironment promotes anti-tumor activity via host adaptive immunity that is T cell-dependent^32^. High doses of intratumoral injection IFN-β largely functioned via an anti-angiogenic effect^33^. IFN-γ can also induce tumor cell apoptosis and exerts an antitumor effect via a tumor necrosis factor-related apoptosis-inducing ligand (TRAIL) pathway, which causes apoptosis in tumor cells by activating caspase-3 with the target cells^20^. Besides IFN-β and IFN-γ, we had also detected up-regulation of cytokines important in innate immune response like IL-2, IL-12, TNF-α, MCP-1 and down-regulation of IL-10 (Figure S3). Our results demonstrated that cGAMP can trigger the production of cytokines including IFN-β and IFN-γ by stimulating antitumor immunity of immunogenic tumors.

### cGAMP Triggers Dendritic Cells Activation

**Figure 4:**
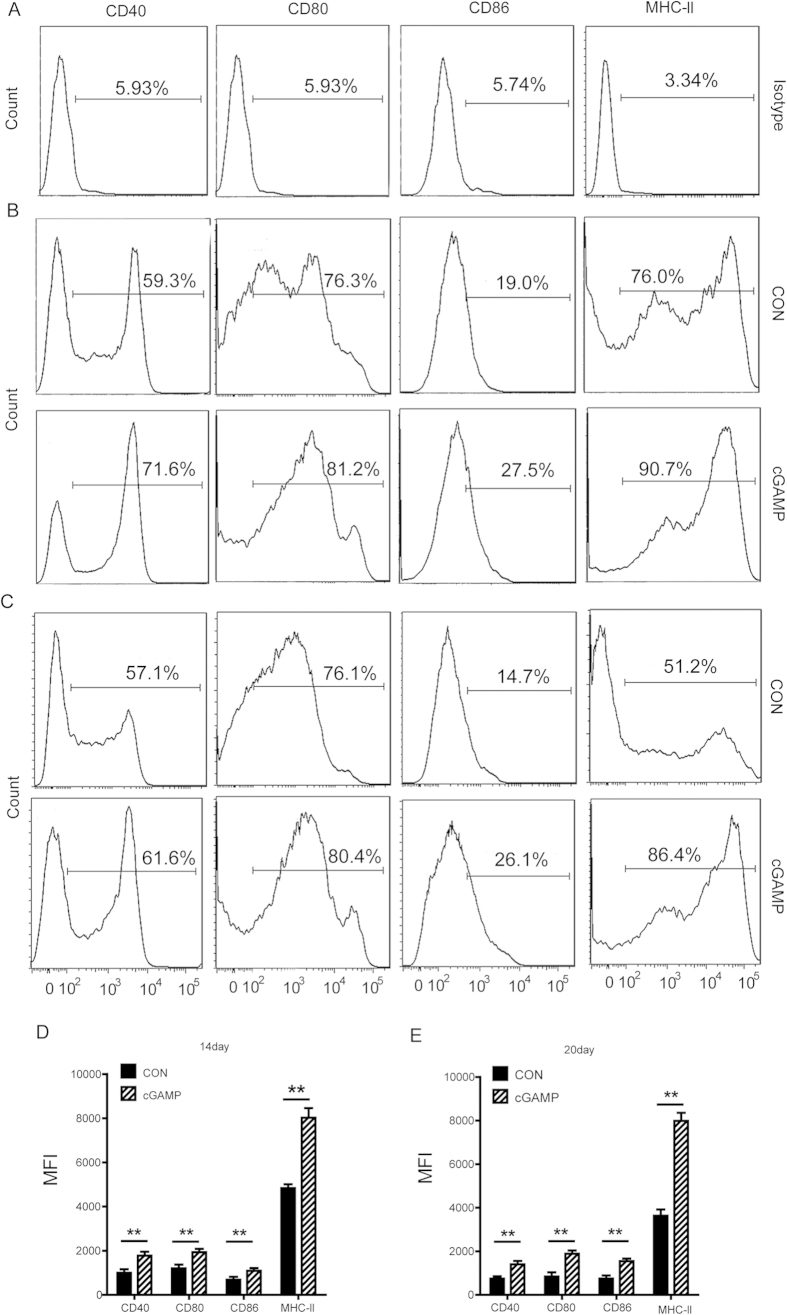
cGAMP Triggers Dendritic Cells Activation. Colon 26 tumor-bearing mice were treated with cGAMP from day 0 to day 20 with 20 mg/kg once a day. Flow cytometry was applied to detect DCs from spleen with (**A**) isotype control, (**B**) key surface markers: CD40^+^, CD80^+^, CD86^+^ and MHC-II^+^ on 14^th^ day and (**C**) 20^th^ day, respectively, and calculated with Mean Fluorescence Intensity (MFI) (**D,E**). Representative data are shown from three experiments conducted with 10 mice per group. Data are represented as mean ± SEM, **p < 0.01 (Student’s t test in **D** and **E**).

Dendritic cells (DCs), serve as antigen-presenting cells specialized to initiate and maintain immunity^34^. Resting DCs are immature and resident in most tissues and can be activated by environmental stimuli to mature into potent APCs. Murine DCs undergo phenotypic maturation upon exposure to type-I IFN *in vivo* or *in vitro*^8^. They play a pivotal role in the initiation, programming, and regulation of immunogenic tumors^4^,^35^. Previous study has shown that cGAMP can directly activate DCs *in vitro*^36^. To investigate whether cGAMP treatment is able to stimulate DCs activation *in vivo*, we evaluated the function of spleen DCs with CD40^+^, CD80^+^, CD86^+^ and MHC-II^+^ by flow cytometry analysis (Fig. 4A–C). As shown in Fig. 4D,E, all CD40^+^, CD80^+^, CD86^+^ and MHC-II^+^ of DCs were significantly up-regulated upon the cGAMP treatment in tumor-bearing mice. cGAMP stimulated and enhanced the antigen-presenting function of DCs, which establishes a potential to activate CD8^+^ T cells. cGAMP triggers DC activation by boosting the expression of IFN-β via the STING-dependent pathway^9^,^19^. IFN-β then induces the expression of the co-stimulatory molecules, such as CD40, CD80, CD86 and the MHC-II, and activate tumor-specific CD8^+^ T lymphocytes^37^,^38^. It has been reported that cGAMP is an adjuvant that stimulates T cell activation in a STING-dependent manner^27^. Consistent with previous study, we also had detected the increase in CD8^+^ T-cell (Figure S4) and up-regulation of related cytokines like IL-2, TNF-α and IFN-γ (Figure S3 & Fig. 3).

### cGAMP Stimulates the Expression of STING and IRF3 in Tumor Tissues

**Figure 5:**
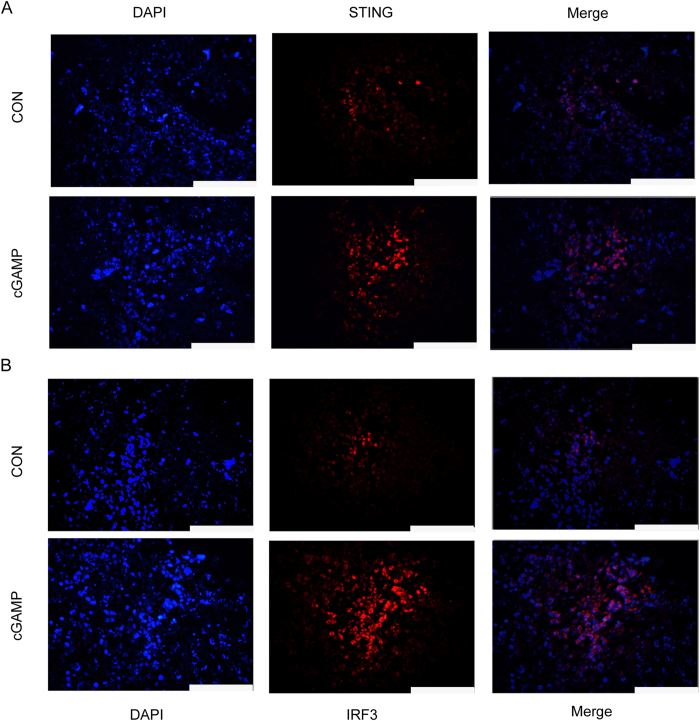
cGAMP Boosts the Expression of STING and IRF3 in Tumor Tissues. Colon 26 tumor-bearing mice were treated with cGAMP from day 0 to day 20 with 20 mg/kg once a day. (**A**) STING (stimulator of interferon genes) and (**B**) IRF3 (IFN regulatory factor 3) were detected with immunofluorescence technique. Scale bar =200 μm.

To further investigate whether cGAMP targets the STING in the cGAS-cGAMP-STING-IRF3 pathway to facilitate antitumor activity, we examined the expression of STING and IRF3 in tumor tissues treated with cGAMP by immunofluorescence assay. As shown in Fig. 5, the expression of both STING and IRF3 in the tumor tissues was obviously up-regulated by cGAMP treatment. The results demonstrated that cGAMP enhanced antitumor immunity in mice via the activation of STING and subsequent stimulation of the cGAS-cGAMP-STING-IRF3 pathway. It has recently been reported that IRF3 activation is part of the first line of defense against invading viruses by inducing the production of IFN-β, and the induced amplification loop of type-I IFN leads to the development of an antiviral state^10^,^22^. We herein found that cGAMP stimulated the production of type-I IFN to exert antitumor effect via stimulating IRF3 activation.

### STING Is Essential for the Antitumor Activity of cGAMP

**Figure 6:**
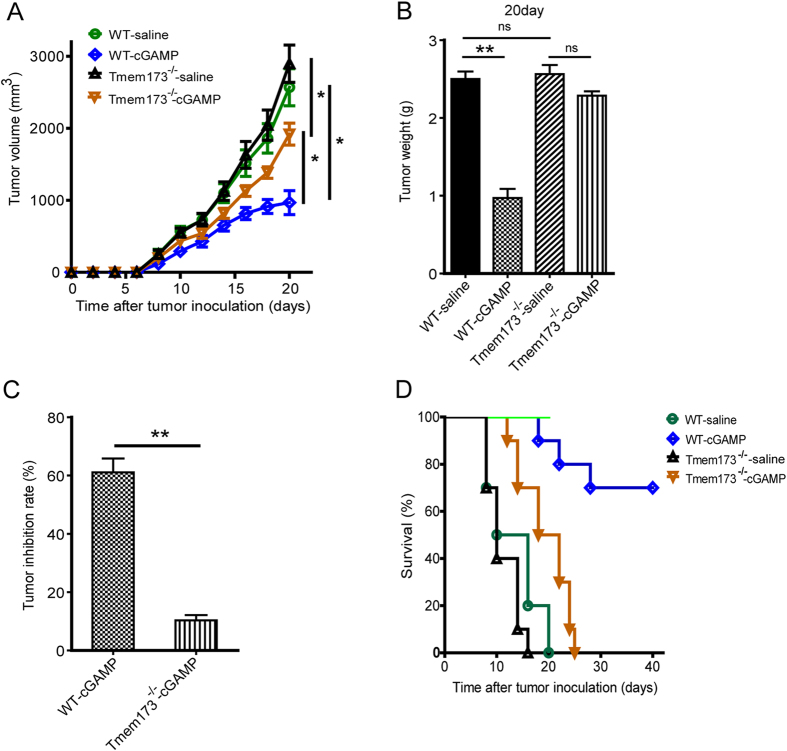
STING Is Essential for the Antitumor Activity of cGAMP. WT and STING^−/−^ (*Tmem173*^−/−^) mice were inoculated and treated with cGAMP (20 mg/kg) for twenty days. (**A**) Mean tumor volumes, (**B**) Tumor weights, (**C**) Tumor inhibition rate, (**D**) Survial of tumor mice were showed. Representative data are shown from three experiments conducted with 10 mice per group. Data are represented as mean ± SEM, *p < 0.05 and **p < 0.01 (Student’s t test).

Given that cGAMP exerts the antitumor effect via stimulating the innate immune pathway of cGAS-cGAMP-STING-IRF3 to enhance production of IFN-β, IFN-γ, as well as to activate DCs, STING is likely targeted directly by cGAMP to stimulate the innate immune response. To test this hypothesis, we conducted antitumor studies of cGAMP in STING^−/−^ mice. We implanted Colon 26 tumor cells on flanks of wild-type (WT) and STING^−/−^ mice (*Tmem173*^−/−^, STING is encoded by *Tmem 173*) and monitored tumor growth upon cGAMP treatment. Our results showed that tumor growth was significantly inhibited by the treatment with cGAMP in WT mice, while the absence of host STING significantly reduced the antitumor effect of cGAMP (Fig. 6). Compared with WT mice, interferon regulatory factors (IRF3) could not be activated in STING^−/−^ mice (Figure S1B). cGAMP treatment could not induce the expression of IFN-β and IFN-γ in STING^−/−^ mice (Figure S5B). As cGAMP triggered dendritic cells activation in wild-type mice, whether cGAMP could still activate DCs in STING^−/−^ mice was investigated. As showed in Figure S6, cGAMP showed no effects on dendritic cells activation in STING^−/−^ mice. These studies demonstrated that STING is essential for the antitumor activity of cGAMP, suggesting that STING-dependent cGAS-cGAMP-STING-IRF3 pathway plays a major role in cGAMP inducing tumor suppression. It is interesting to note that cGAMP still have some tumor suppressive activity in STING^−/−^ mice (Fig. 6), indicating cGAMP may stimulate other STING independent pathways to suppress tumor growth when STING is absent.

### Combination of cGAMP and 5-FU Improve the Antitumor Activity and Reduce the Toxicity of 5-FU Chemotherapy

**Figure 7:**
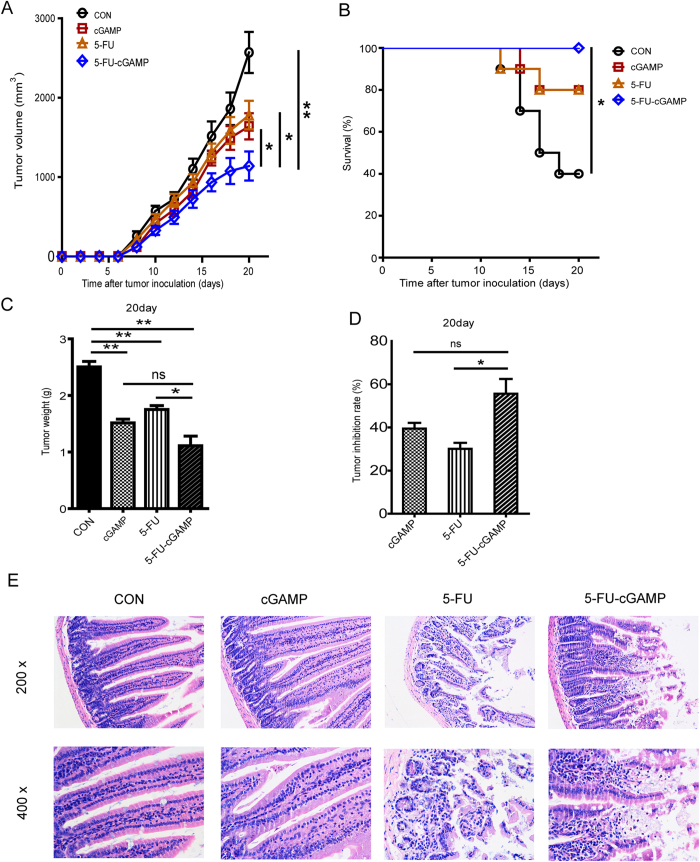
cGAMP with 5-FU Improve the Antitumor Activity and Reduce the Toxicity of 5-FU Chemotherapy. Colon 26 tumor-bearing mice were treated with cGAMP (5 mg/kg) and 5-FU (10 mg/kg) once a day. (**A**) Mean tumor volumes, (**B**) Survival rate of mice for twenty days. (**C**) Tumor weights. (**D**) Tumor inhibition rate. (**E**) Hematoxylin-Eosin staining for detection of small intestine tissues. Representative data are shown from three experiments conducted with 10 mice per group. Data are represented as mean ± SEM, *p < 0.05 and **p < 0.01 (Student’s t test in **A**, **C** and **D**, and log rank [Mantel-Cox] test in **B**).

Combination of multiple drugs has showed better therapeutic efficacy in antitumor treatment. To test if cGAMP can boost the antitumor activity of 5-FU and reduce 5-FU-induced toxic side effects, we examined the antitumor activity of cGAMP in combination with 5-FU. We found that, with the combination of 5-FU and cGAMP treatment, tumor growth in mice (Fig. 7A,C,D) was inhibited much more effectively, and survival rate (Fig. 7B) was increased from 80% (treated with 5-FU alone) to 100% (treated with 5-FU plus cGAMP). Chemotherapy-induced mucosal barrier dysfunction can lead to nausea, vomiting, diarrhea, severe mucosal necrosis, blood in the stool and systemic adverse reactions, and even lead to disease progress^39^. The side-effects of 5-FU result in intestinal atrophy, as indicated by increased intestinal permeability and shorter villi, disorders in intestinal flora^40^,^41^. As shown in Fig. 7E, HE staining assay for small intestine structure showed that cGAMP does not induce obvious toxicity to intestinal tissue. The small intestine showed the same microstructure in cGAMP group as those in un-treated control group (Fig. 7E, leftmost panels); while 5-FU-treated group showed severe damage of the small intestinal epithelial tissue (Fig. 7E, the panels next to rightmost panels), resulting in mucosal atrophy. Interestingly, when the tumor-bearing mice were treated with 5-FU in combination with cGAMP, the damage of intestinal mucosal epithelial tissue was alleviated significantly (Fig. 7E, right most panels). Taken together, these studies have shown that cGAMP not only improve the antitumor activity of 5-FU significantly, but also reduce the toxicity of 5-FU, when using a combination therapy.

## Discussion

Immunotherapy is revolutionizing the treatment of cancer patients. Great progresses have been recently made in understanding the structural basis and molecular mechanism of the newly discovered cGAS-cGAMP-STING-IRF3 pathway in innate immunity^21^,^22^,^23^,^24^,^25^,^26^. cGAS is an innate immune sensor of HIV and other retroviruses, and cGAS-cGAMP-STING-IRF3 signaling plays a pivotal role in antiviral defense and immune adjuvant effects^22^,^27^,^30^. cGAMP is a small second messenger molecule (675 Da) that binds to STING and triggers the innate immune responses for antiviral defense. Recently, more and more studies show STING mediated immune response is also critical in regulating antitumor immunity^18^,^37^,^42^. Direct activation of STING might provide a new strategy to improve cancer therapies. STING agonists such as DMXAA (5, 6-dimethylxanthenone-4-acetic acid), c-di-GMP and cGAMP could provide a new therapeutic strategy.

In this study, we systematically studied the antitumor activity of cGAMP against murine Colon 26 adenocarcinoma with mouse models. Our results showed that cGAMP has potent antitumor activity, which is dose dependent. When used at the dosage of 20 mg/kg, the tumor inhibition rate at the 20^th^ day was over 60%. The survival rate of WT tumor-bearing mice treated daily with cGAMP was still 70% for 40 days. In addition, in established tumor model, cGAMP still showed antitumor activity and the tumor inhibition rate was over 40% for 20 days cGAMP treatment (Figure S7). TUNEL assay and HE staining analysis of tumor tissues showed that cGAMP induced tumor cell nuclei lysis, apoptosis, and severe tumor tissue damage. To investigate whether cGAMP can kill tumor cells directly, cytotoxicity of cGAMP on CT26 cells was performed by MTT assay. The results indicated that cGAMP did not show obvious toxicity against CT26 cells. Therefore, cGAMP can’t inhibit the growth of tumor cell directly. We suppose that cGAMP exerts antitumor effects through boosting the STING innate immune signaling pathway.

DCs are ideal antigen-presenting cells and can induce the cross-priming of CD8^+^ T cells^8^. Type-I IFN exert multiple effects on DCs, affecting the major cellular pathways associated to their antigen-presenting cell (APC) function, namely differentiation, maturation, and migration and up-regulation of MHC-I, CD40, CD80, CD86, and CD83 molecules resulting in a superior capacity to induce CD8^+^ T-cell responses^18^,^43^,^44^,^45^. Type-I IFNs, as stimulators of DC-mediated cross-priming have critical impact on antitumor response, and are the prototype inflammatory cytokines released upon stimulation of the cGAS-cGAMP-STING-IRF3 immune pathway^8^,^14^. Therefore, DC-mediated cross-priming is crucial for antitumor immunity^46^.

In this study, analysis of host serum (by ELISA) and tumor tissue (by Real-time PCR) showed that cGAMP significantly up-regulated antitumor cytokines, such as IFN-β and IFN-γ. DCs from the spleen were activated by cGAMP, as assessed by flow cytometry with CD40^+^, CD80^+^, CD86^+^ and MHC-II^+^. These results demonstrated that cGAMP stimulated DCs activation, and boosted the innate immune response of immunogenic tumors. Tumor site analysis from immunofluorescence detection indicated that STING and IRF3 were significantly up-regulated upon cGAMP treatment. Whereas, the absence of host STING dramatically reduced the antitumor effect of cGAMP. These results provide evidence that using cGAMP stimulates and enhances the innate immune response through the cGAS-cGAMP-STING-IRF3 pathway, and increases antitumor cytokines production and DCs activation to inhibit tumor growth. As an endogenous second messenger in innate immune signaling by cytosolic DNA, cGAMP binds to STING and activates IRF3 in a STING-dependent manner and induce IFN-β production^14^. Endogenously produced IFN-β is critical for the induction of an antitumor immune response resulting in the elimination of tumors^33^.

Many kinds of cancers appear to induce a spontaneous adaptive T cell response. Targeting low doses of type I IFNs to the tumor microenvironment also promotes anti-tumor activity via host adaptive immunity that is T cell-dependent^8^,^16^. The presence of T cell infiltrate has been linked to favorable clinical outcome in multiple types of cancers^47^,^48^. However, the innate immune pathways that bridge to an adaptive immune response under sterile conditions are poorly understood. This study revealed that cGAMP can induce IFN-β, IFN-γ production and DCs activation, which are required for a spontaneous T cell response *in vivo*. DCs secrete and respond to type-I IFN as autocrine DC activators^38^,^45^. Basal expression of type-I IFN by DCs leads to secretion of IFN-γ, which may in turn act to enhance DC activation^38^.

cGAMP can activate STING-IRF3 signaling pathway and stimulate IFN-γ expression^9^,^10^,^11^,^12^,^13^. In mPBMCs, synergistic production of innate IFN-γ was completely dependent on IRF3 and IRF7, which are required for the induction of type-I IFNs, and STING signaling pathway is response for type-I IFNs^49^. IFN-γ is a key cytokine in tumor immunology. IFN-γ is produced predominantly by T lymphocytes, NKT cells and natural killer (NK) cells^50^,^51^. IFN-γ is essential for rejection of transplanted tumor cells, destroys existing tumor stroma and decreases tumor development^52^.

Besides IFN-β and IFN-γ, cGAMP could regulate the expression of other cytokines (Figure S3). In tumor-bearing mice, IL-2, IL-6, IL-12, TNF-α, and MCP-1 were up-regulated while IL-10 was down-regulated after cGAMP treatment. IL-2 has been approved by the Food and Drug Administration (FDA) for the treatment of cancers. IL-6 is a pleiotropic cytokine secreted by many different cells, including the monocyte/macrophages, mast cells, T cells, and DCs. IL-6 influences antigen-specific immune responses and inflammatory reactions^53^. Just as Fisher DT *et al.* reviewed the two faces of IL-6 in the tumor microenvironment, while IL-6 signaling is generally considered as a key driver of malignancy, accumulating evidence establishes IL-6 is a key player in the mobilization of anti-tumor T cell responses^54^. IL-6 signaling can also resculpt the T cell immune response, shifting it from a suppressive to a responsive state that can effectively act against tumors. IL-6 plays an indispensable role in boosting CD8^+^ T cell trafficking to tumor sites, where they have the opportunity to become activated and execute their cytotoxic effector functions, respectively^54^. IL-12 can act as a growth factor for activated T and NK cells, enhance the lytic activity of NK cells, and stimulate the production of IFN-γ by resting PBMC^55^. IL-12 regulates VEGF and MMP to exert potent anti-angiogenic effects which contribute to tumor regression in cancer model^56^. In addition, IL-12 with IFN-γ are important cytokines for tumor surveillance^57^. The role of MCP-1 in angiogenesis and tumor progression has been revealed, the expression level of MCP-1 is correlated with anti-tumor activity^58^,^59^. IL-10 suppresses anti-tumor immunity while T cell-derived IL-10 promotes cancer growth by suppressing both T cell and APC function^60^. Based on the roles of these cytokines in anti-tumor immunity, the anti-tumor activity of cGAMP might be related to regulation of these cytokines, although how cGAMP tunes the protein expression levels of some cytokines is still unclear.

DMXAA, a vascular disrupting agent, can activate dendritic cells and possess strong anti-tumor activity in mouse models^61^,^62^. DMXAA binds mouse mSTING but not human hSTING, and has failed in clinical trials in non-small-cell lung cancer^26^,^63^. To evaluate safety of cGAMP, toxicity of cGAMP treatment was investigated. The toxicity of liver and kidney tissues induced by cGAMP was also examined by HE staining, and we found that cGAMP had no toxicity to liver and kidney (Figure S8). In addition, cGAMP treatment has no direct impact on T cells (Figure S9). As a natural ligand for hSTING, cGAMP might be more promising in antitumor drug design and discovery compared to DMXAA.

5-FU is a clinical antitumor drug with interference of DNA synthesis with obvious side-effects. According to NCCN guidelines in colon cancer (Version 1, 2014), adjuvant therapy with 5-FU alone or combination is not only recommended by the panel for patients with stage III disease but also for patients with high-risk stage II disease. Immunotherapeutic strategies maybe enhance the response to anticancer therapies. Here we found that cGAMP, as a stimulator/enhancer of innate immune antitumor agent, can not only improve the antitumor effects of 5-FU, but also attenuate the 5-FU-induced side-effects. The possible mechanism of cGAMP can reduce cytotoxicity of 5-FU maybe related to dendritic cells. Tadagavadi, R.K. *et al.* reported dendritic cells ameliorated cisplatin-induced nephrotoxic acute kidney injury and their protection from cisplatin nephrotoxicity was independent of neutrophils^64^,^65^. cGAMP can enhance innate immune responses by inducing production of cytokines and stimulating dendritic cells activation, which may be able to reduces 5-FU-induced cytotoxicity. cGAMP alone is more suitable to patients with early stage colon cancer or prevent tumor recurrence or metastasis after chemotherapy or operation. The combination cGAMP with chemotherapy can supply to patients with high-risk stage II or stage III disease.

In summary, we have conducted the first systematic studies of the antitumor activity of cGAMP using mice models and its molecular mechanism against murine Colon 26 adenocarcinoma has been elucidated. Our results revealed that cGAMP has potent antitumor activity in mice through cGAS-cGAMP-STING-IRF3 pathway. Moreover, cGAMP can not only improve the antitumor activity of 5-FU, but also clearly reduce the toxicity of 5-FU. These findings in the current study strongly suggest that cGAMP is likely to be potent for the treatment of colorectal cancer and demonstrated that the direct stimulation of the innate immune response by cGAMP has potential application in immunotherapy of cancer.

## Methods

### Preparation of cGAS and cGAMP

Mouse cGAS protein and cGAMP were prepared as described^21^,^22^. Briefly, mouse cGAS was cloned into a modified pET-28(a) vector with an N-terminal SUMO tag. The protein was expressed in *E. coli* BL21 (DE3) at 15 °C and purified by Ni-NTA and Superdex 200 gel filtration columns as described^10^,^21^. cGAMP was synthesized and separated as follows: cGAS was incubated with DNA in a reaction buffer containing 20 mM HEPES (pH 7.5), 5 mM MgCl_2_, 2 mM ATP, and 2 mM GTP at 37 °C for 2 hours. The samples were centrifuged, passed through a 10 kD ultrafiltration filter (Millipore), analyzed and purified by ion exchange chromatography using a MonoQ column (GE Healthcare). cGAMP was further purified through endotoxin removal kit (Thermo Fisher Scientific). Endotoxin level was tested using Pierce LAL Chromogenic Endotoxin Quantitation Kit (Thermo Fisher Scientific). Endotoxin-free cGAMP was lyophilized and stored at −20 °C.

### Mice and Cells

BALB/c mice (body weight: 20~25g) were obtained from SIPPR-BK Experimental Animal Co. (Shanghai, China). *Tmem173*^−/−^ (STING-deficient) mice were purchased from the Jackson Laboratory in California USA. All the mice were maintained under specific pathogen-free conditions and used according to the animal experimental guidelines set by National Institutes of Health Guide for the Care and Use of Laboratory Animals. This study has been approved by the Institutional Animal Care and Use Committee of Fudan University and the Scientific Investigation Board of Second Military Medical University, Shanghai. All animal experiments were carried out in accordance with established International Guiding Principles for Animal Research. Colon 26 cells were cultivated in Dulbecco’s modified eagle media (Hyclone, USA), supplemented with 10% heat-inactivated fetal bovine serum (GIBCO, USA) containing penicillin/streptomycin at 37 °C under 5% CO_2_ and 95% humidity incubator.

### Tumor Growth and Treatments of Mice Models

Colon 26 adenocarcinioma cells (1 × 10^6^) were injected subcutaneously into the right flank of mice. Mice were divided into 5 groups: (a) Control group with tumor cells injection; (b, c, d) tumor groups with tumor cells injection and treated with cGAMP at various dosages of 5, 10 and 20 mg/kg, respectively; (e) *Tmem173*^−/−^ (STING-deficient) mice group, with injection of tumor cells and treated with cGAMP (10 mg/kg). Four hours after injection of tumor cells, cGAMP was intravenously injected into the tumor-bearing mice daily for 20 days. Or cGAMP was intravenously injected daily after tumors were established. Tumors were measured and the weight of tumor-bearing mice was checked daily. On day 20^th^, all mice were weighed and sacrificed, and all the tumors were removed and weighed. According to the mean weight of tumors, the tumor inhibition rate was calculated as follows: the tumor inhibition rate (%) = [(mean tumor weight of control group − mean tumor weight of tumor group with cGAMP-treatments)/mean tumor weight of control group] × 100%.

### ELISA

Mice blood serum samples were collected at ophthalmic vein and kept at room temperature for 2 h before centrifugation for 20 minutes at 1000g. The assay serum was freshly prepared. Concentration of IFN-β, IFN-γ, IL-2, IL-6, IL-10, IL-12, MCP-1 and TNF-α were measured with mouse ELISA kits (R&D Systems) according to the manufacture’s instruction, respectively.

### Spleen Cell Isolation and Flow Cytometry

Spleen cells were isolated with the method described^66^. Briefly, murine spleen were disrupted and filtered through 40 μm strainer to obtain single-cell suspensions. Splenocytes were resuspended in PBS buffer containing 0.5% BSA, and red blood cells were lysed with ACK lying buffer. Splenic CD11c^+^ DCs were labeled by CD11c mAb microbeads and isolated with MACS separation column (Miltenyi Biotec, Auburn, CA). Monoclonal antibodies including anti-mouse CD40, anti-mouse CD80, anti-mouse CD86 and anti-mouse MHC Class II were used for DCs detection. Purified DCs were incubated with antibody, respectively. For CD8^+^ T cells detection, splenic CD3^+^ CD8^+^ T cells were stained with both CD3 antibody and CD8α antibody. Monoclonal antibodies used for flow cytometry were all purchased from eBiosciences (San Diego, CA) and Flow cytometric analysis was performed on the BD FACS Cablibur flow cytometer (BD Bioscience, San Jose, CA).

### RNA Extraction and Quantitative Real-Time PCR

Total RNA was purified from tumor tissues using TRIzol Reagent (Takara, Japan) and reversed-transcribed to cDNA with Prime Script TM RT Master Mix (Takara, Japan) according to manufacturer’s instructions. Real-time PCR was performed with Applied Biosystems 7500 (Life Technologies Corporations, Carlsbad, CA, USA) with initial denaturation at 95 °C for 30 s, and followed by 40 cycles of denaturation (95 °C, 5 s), annealing (55 °C, 30 s), and extension (72 °C, 30 s). Relative expression changes were calculated with ΔΔCt method. GAPDH served as the internal control. The primer sequences are as follows:

GAPDH: Forward: 5′-CCAGCCCAGCAAGGATACTG-3′

Reverse: 5′-GGTATTCGAGAGAAGGGAGGGC-3′

IFN-β: Forward: 5′-TCCGAGCAGAGATCTTCAGGAA-3′

Reverse: 5′-TGCAACCACCACTCATTCTGAG-3′

IFN-γ: Forward: 5′-AGCAACAGCAAGGCGAAAA-3′

Reverse: 5′-CTGGACCTGTGGGTTGTTGA-3′

IRF3: Forward: 5′-AACCGTGGACTTGCACATCT-3′

Reverse: 5′-GCCATGCTGTGTTTTGTCCC-3′

### Immunofluorescence

Tumor tissues were rapidly excised, rinsed with PBS buffer and fixed in 4% paraformaldehyde. 5 μm thick paraffin-embedded tumor sections were cut and deparaffinized in xylene and rehydrated in ethanol. The slides were blocked with 5% BSA, stained with primary antibody Tmem173 (Abcam, ab92605), IRF3 (Abcam, ab68481) or CD45 (Abcam, ab64100) with the dilution of 1:100, respectively, then stained with secondary antibody at 1:100, respectively. Then the slides were washed with PBS buffer and photos were taken with fluorescence microscope (Leica, Germany).

### TUNEL Assay of Tumor Cells Apoptosis

Apoptotic tumor cells were determined by terminal deoxynucleotidyl transferase-mediated deoxyuridine triphosphate nick end labeling (TUNEL) staining (In Suit Cell Death Detection Kit, Fluorescein, Roche). For TUNEL assay, the slides were heated at 60 °C followed by washing in xylene and rehydration through a graded series of ethanol. Tissue sections with proteinase K were incubated in working solution for 20 minutes, followed by TUNEL reaction mixture containing label and enzyme solution, with slide incubated in the dark. After rinsing the slide with PBS buffer, samples were analyzed under fluorescence microscope.

### HE Staining

Tumor tissues, liver and kidney tissues were fixed in 4% paraformaldehyde for 24 h. 5 μm thick section of tumor tissues were mounted on the slide and deparaffinized, and then stained with hematoxylin and Eosin (HE). The section was examined under light microscope (Leica, Germany)

### MTT assay

CT26 cells were plated in 96-well culture plates at 5 × 10^4^ cells/well, and incubated with cGAMP (10-200 μM) for 48 h. Cell viability assay were performed using MTT method. MTT was added at a final concentration of 5 mg/mL, after incubation at 37 °C for 4 h, the medium was removed and 150 μL DMSO were added per well. The absorbance was measured at 490 nm.

### CCK-8 assay

T Cells were purified with microbeads (MagniSort Mouse T cell Enrichment Kit, eBioscience) from spleen according to the manufacturer’s instructions. Cells were plated in 96-well culture plates and incubated with cGAMP (10-200 μM) for 48 h. 10 μL CCK-8 solution was added to each well and cell viability was measured by a microplate reader at 450 nm.

## Additional Information

**How to cite this article**: Li, T. *et al.* Antitumor Activity of cGAMP *via* Stimulation of cGAS-cGAMP-STING-IRF3 Mediated Innate Immune Response. *Sci. Rep.* **6**, 19049; doi: 10.1038/srep19049 (2016).

## Supplementary Material

Supplementary Information
