# Supplementary material for: Antitumor Activity of cGAMP via Stimulation of cGAS-cGAMP-STING-IRF3 Mediated Innate Immune Response
Source: Sci Rep. 2016 Jan 12;6:19049. doi: 10.1038/srep19049 (PMC4709567; doi:10.1038/srep19049)
Supplement: Supplementary Information [file srep19049-s1.pdf]

Supplementary Information for

Antitumor Activity of cGAMP *via* Stimulation of cGAS-cGAMP-STING-IRF3 Mediated Innate Immune Response

Tiejun Li [2,+], Hao Cheng[1,+], Hong Yuan[1,+], Qiming Xu[1], Chang Su[3], Yuefan Zhang[2], Pengbiao Xu[1], Jason Tan[3], Yaocheng Rui[2], Pingwei Li[3], and Xiangshi Tan[1*]

[1]Department of Chemistry & Shanghai Key Laboratory of Chemical Biology for Protein Research and Institutes of Biomedical Sciences, Fudan University, Shanghai, 200433, China

[2]Department of Pharmacology, School of Pharmacy, Second Military Medical University, Shanghai, 200433, China

[3]Department of Biochemistry and Biophysics, Texas A&M University, College Station, TX 77843, USA

Figure S1. cGAMP could not stimulate the expression of IRF3, IFN-β and IFN-γ in STING defective mice

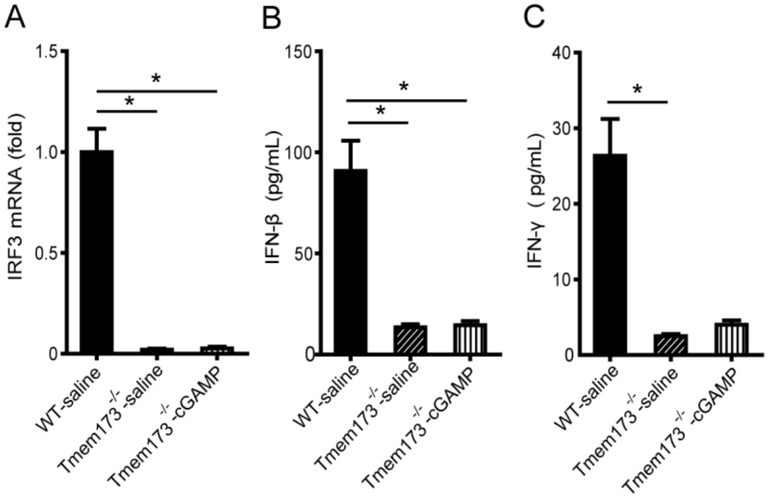

STING$^{-/-}$ mice were treated with cGAMP (20 mg/kg) once a day. (A) The relative expression of IRF3 was detected by Real-time PCR in tumor tissues. (B) The concentration of IFN-β and IFN-γ in serum was measured by ELISA. Representative data are shown from two experiments conducted with 10 mice per group. Data are represented as mean ± SEM.

Figure S2. cGAMP has no direct impact on CT26 cells and T cells in vitro

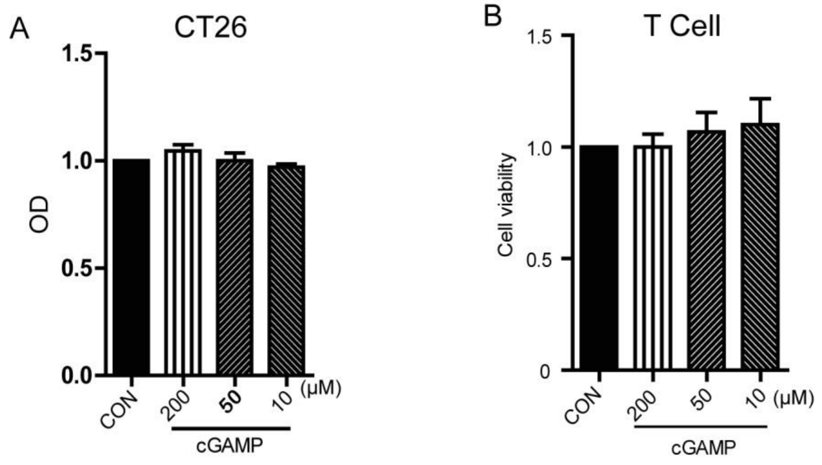

CT26 cells were plated in 96-well culture plates at 5×10^4 cells/well, and incubated with cGAMP for 48h. CT26 cell viability were analyzed by MTT assay. T cells were purified with microbeads from spleen according to the manufacturer's instructions. Cells were plated in 96-well culture plates and incubated with cGAMP for 48h. Viability of T cells were analyzed by CCK8 assay cGAMP has no cytotoxicity on (A) CT26 cells, (B) T cells.

Figure S3. cGAMP has no impact on immune cells

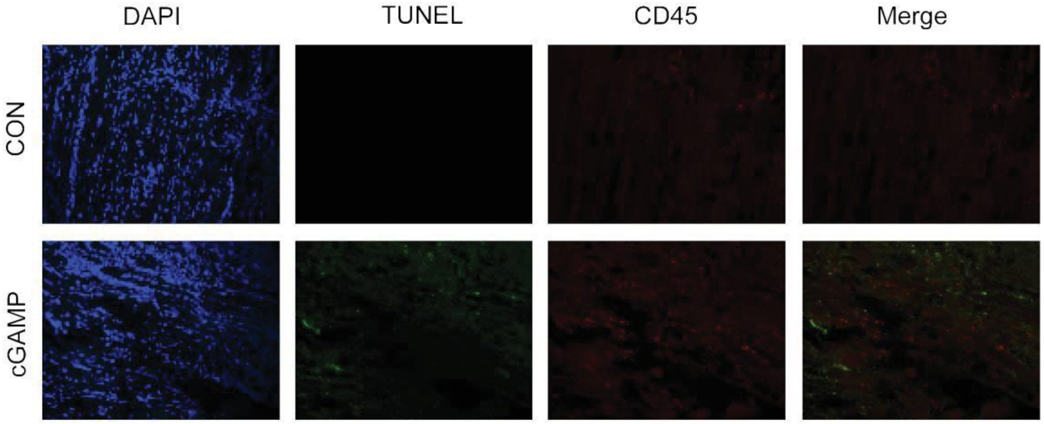

Colon 26 tumor-bearing mice were treated with cGAMP from day 0 to day 20 with 20 mg/kg daily. TUNEL and CD45 were detected with tumor tissues by immunofluorescence technique (200×).

Figure S4. cGAMP can't active DCs in STING defective mice

A

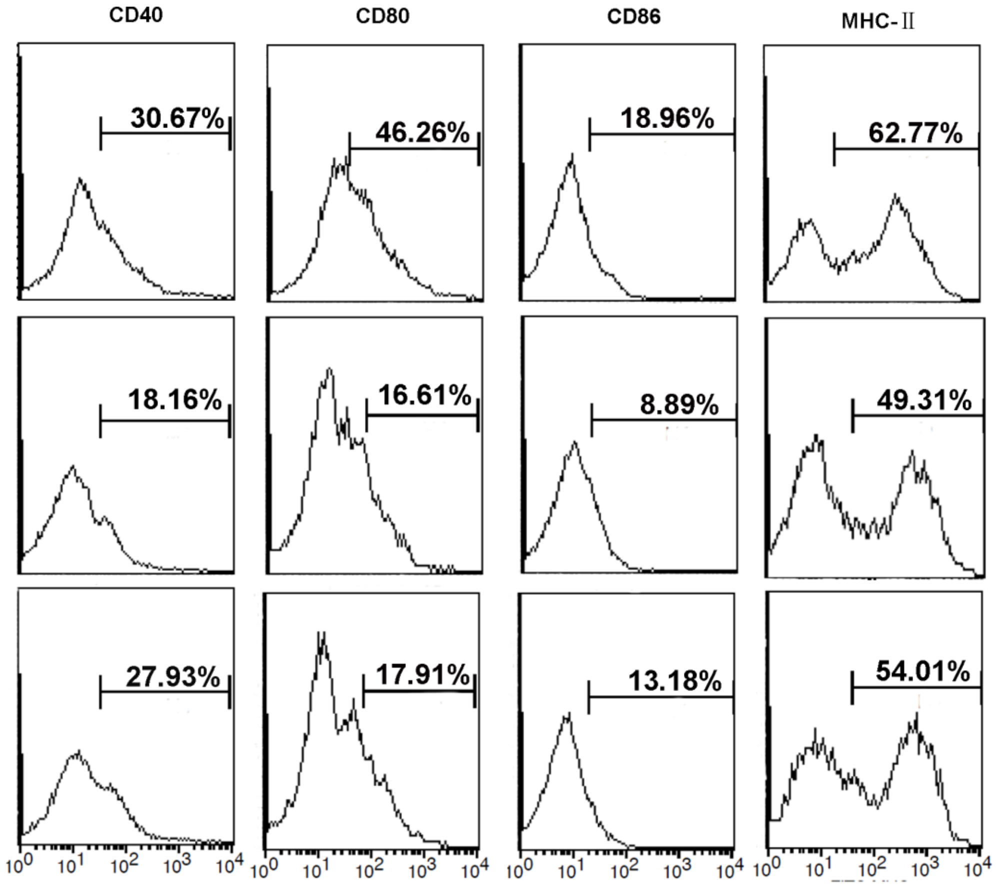

B

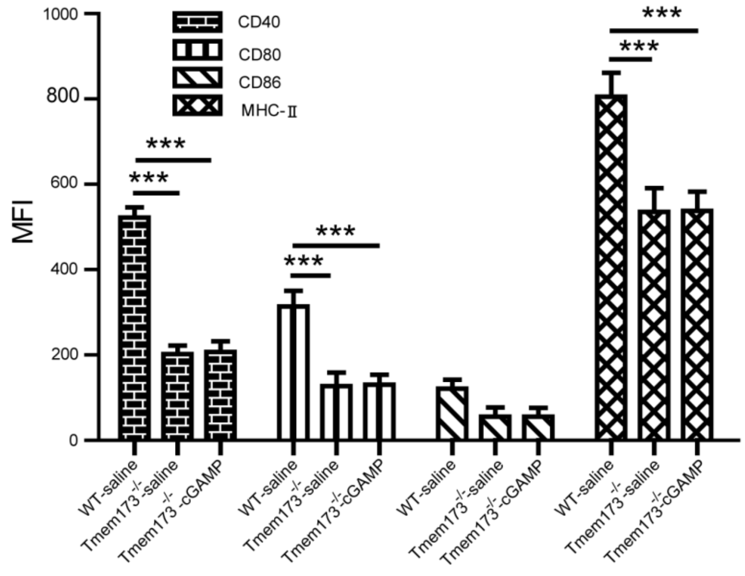

Tumor bearing wild-type and STING$^{-/-}$ mice were treated with or without cGAMP from day 0 to day 20 with 20 mg/kg daily. Flow cytometry was applied to detect DCs from spleen with (A) key surface markers: CD40^{+}, CD80^{+}, CD86^{+} and MHC-II on 20th day, respectively, and calculated with (B) Mean Fluorescence Intensity (MFI). Representative data are shown from two experiments conducted with 10 mice per group. Data are represented as mean ± SEM.

Figure S5. cGAMP induces increase of $CD8^+$ T cells

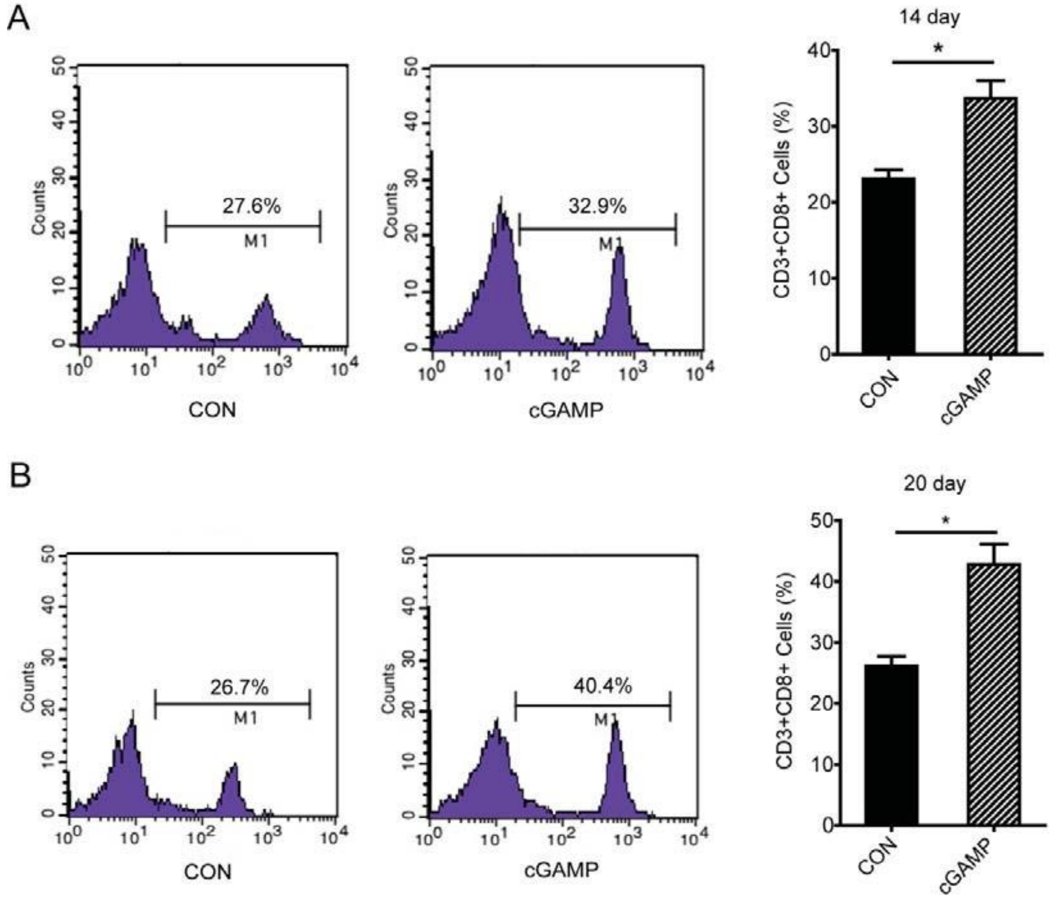

Colon 26 tumor-bearing mice were treated with cGAMP from day 0 to day 20 with 20 mg/kg daily. Flow cytometry was applied to detect $CD3^+CD8^+$ T cells from spleen with key surface markers: $CD8^+$ on 14th day (A) and 20th day (B) respectively, and calculated with percentage of CD8 positive cells in $CD3^+$ T cells (A and B). Representative data are shown from two experiments conducted with 10 mice per group. Data are represented as mean ± SEM, *$p < 0.05$ (Student's t test in A and B).

Figure S6. cGAMP has no toxicity on liver and kidney tissues

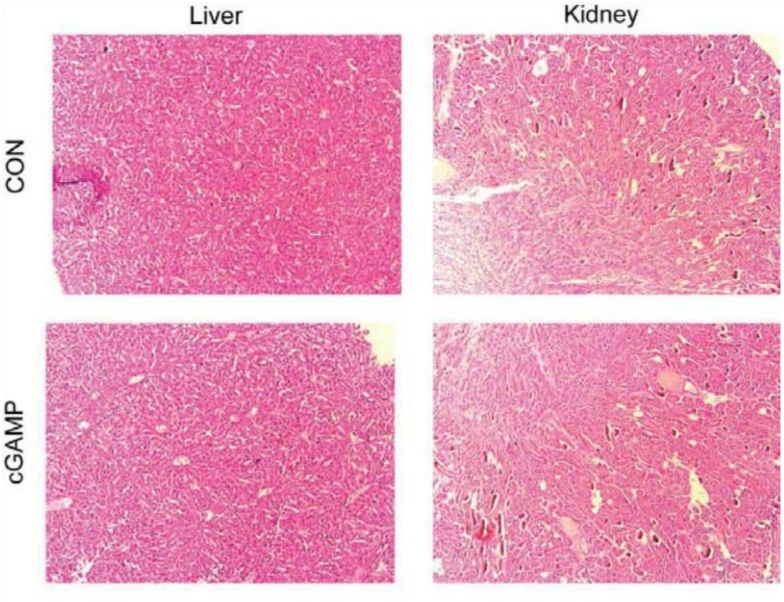

Colon 26 tumor-bearing mice were treated with cGAMP (20 mg/kg) daily for 20d. The liver and kidney tissues were embedded and HE staining was carried out for pathological detection (200×).

Figure S7. cGAMP regulates expression of cytokines in tumor bearing mice

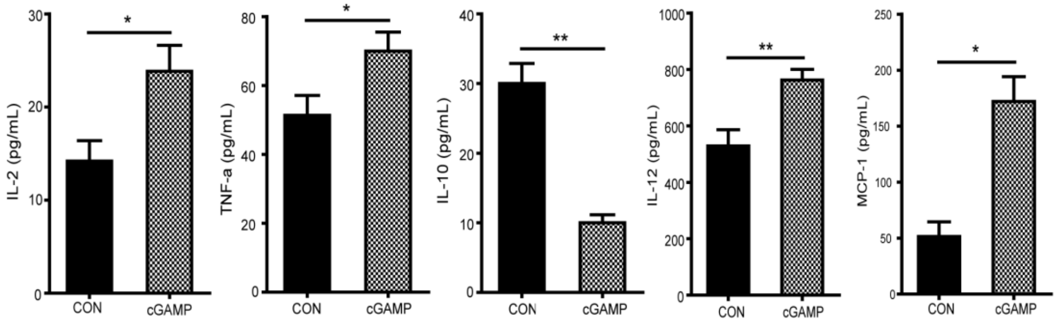

Tumor-bearing mice were treated with cGAMP (20 mg/kg) daily. Concentration of cytokines in serum was measured by ELISA. Representative data are shown from two experiments conducted with 10 mice per group. Data are represented as mean ± SEM.

Figure S8. cGAMP possesses anti-tumor activity in established colon 26 adenocarcinoma tumor model

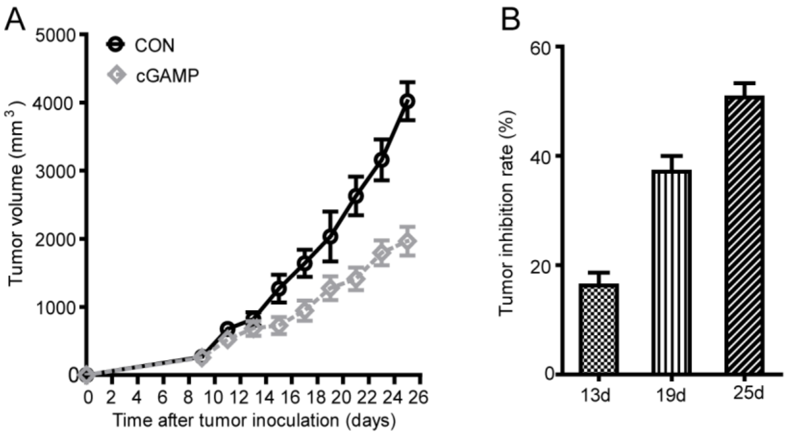

cGAMP was intravenously injected daily after tumors were established (9 days after tumor cells implantation). Colon 26 tumor-bearing mice were treated with cGAMP from day 9 to day 26 with 20 mg/kg. (A) Mean tumor volumes. (B) Tumor inhibition rates of mice in different days.
